# Estimating County-Level Mortality Rates Using Highly Censored Data From CDC WONDER

**DOI:** 10.5888/pcd16.180441

**Published:** 2019-06-13

**Authors:** Harrison Quick

## Abstract

**Introduction:**

CDC WONDER is a system developed to promote information-driven decision making and provide access to detailed public health information to the general public. Although CDC WONDER contains a wealth of data, any counts fewer than 10 are suppressed for confidentiality reasons, resulting in left-censored data. The objective of this analysis was to describe methods for the analysis of highly censored data.

**Methods:**

A substitution approach was compared with 1) a simple, nonspatial Bayesian model that smooths rates toward their statewide averages and 2) a more complex Bayesian model that accounts for spatial and between-age sources of dependence. Age group–specific county-level data on heart disease mortality were used for the comparisons.

**Results:**

Although the substitution and nonspatial approach provided age-standardized rate estimates that were more highly correlated with the true rate estimates, the estimates from the spatial Bayesian model provided a superior compromise between goodness-of-fit and model complexity, as measured by the deviance information criterion. In addition, the spatial Bayesian model provided rate estimates with greater precision than the nonspatial approach; in contrast, the substitution approach did not provide estimates of uncertainty.

**Conclusion:**

Because of the ability to account for multiple sources of dependence and the flexibility to include covariate information, the use of spatial Bayesian models should be considered when analyzing highly censored data from CDC WONDER.

**Summary**

**What is already known on this topic?**

Ignoring the impact of suppression due to small counts leads to biased inference.

**What is added by this report?**

This work describes and compares multiple approaches for analyzing highly suppressed data from CDC WONDER. R and WinBUGS code are provided to conduct the analyses.

**What are the implications for public health practice?**

The use of spatial Bayesian models can yield improved inference from the analysis of highly suppressed data such as those available on CDC WONDER.

## Introduction

CDC WONDER (Wide-ranging ONline Data for Epidemiologic Research) is a system developed by the Centers for Disease Control and Prevention (CDC) to promote information-driven decision making by public health practitioners and researchers and provide access to detailed public health information to the general public (1). Although CDC WONDER contains a wealth of data, it has limitations. Per CDC policy (2), any counts fewer than 10 should be suppressed for confidentiality reasons, resulting in left-censored data. Because of high rates of suppression, many chronic disease researchers opt to focus their inference in a few highly populated regions (3) or state- or national-level trends (4), despite known geographic disparities in many chronic disease outcomes (5,6). This suppression may also discourage research on disparities between subsets of the population (eg, race or sex disparities) to avoid reducing already small counts below suppression thresholds. In short, suppression of small counts exacerbates many issues commonly encountered in the field of small area estimation, where the term “small area” refers to a geographic scale (eg, county, census tract) at which the observed data alone do not provide reliable inference. Thus, when CDC WONDER data are used to conduct surveillance, the ability to estimate rates for rural areas and minority populations — where the chronic disease burden is high (7) — is significantly hindered by data suppression.

To address CDC WONDER’s data suppression issue, Tiwari et al (8) proposed an algorithm for estimating age-standardized rates in which suppressed age-specific counts are replaced with estimates based on the county’s age-specific population size and the state-wide average rate for that age-group. For example, suppose *y_ik_* denotes the number of deaths from age-bracket *k* in county *i* of a population of size *n_ik_* and our inferential interest lies in λ*_ik_*, the corresponding mortality rate. Tiwari et al (8) proposed replacing the suppressed *y_ik_* <10 with $y_{ik}^{*}={\overset{-}{\lambda }}_{s_{i}0k}\times n_{ik}$, where *s_i_* denotes the state that county *i* belongs to and ${\overset{-}{\lambda }}_{s_{i}0k}$ denotes the state-wide average rate for age-bracket *k* in state *s_i_* such that

${\overset{-}{\lambda }}_{s_{i}0k}=\sum_{j:s_{j}\;=\;s_{i}}y_{jk}/\sum_{j:s_{j\;}=\;s_{i}}n_{jk}$ (Equation 1)

Because state-level totals are often 10 or greater, we will assume from this point forward that ${\overset{-}{\lambda }}_{s_{i}0k}$ is known and publicly available; when this is not the case, rates could be smoothed toward an alternative value (eg, national estimates).

Although this approach may yield reasonable estimates, it has drawbacks. First and foremost, estimating the uncertainty in age-standardized rate estimates is not an exact science when the data are known (9,10), much less when the data are highly suppressed. Furthermore, the algorithm is not designed to account for heterogeneity in demographic information such as the racial/ethnic make-up and socioeconomic status of the counties’ populations. As a result, inference based on these substituted data may be both biased (ie, smoothing toward the wrong values) and too precise (ignoring the uncertainty due to data suppression).

When the goal is to assess geographic disparities in age-standardized rates between regions, overcoming the privacy protections to obtain trustworthy estimates of the age-specific rates and their levels of uncertainty is only half the battle. For instance, Fay (11) followed the work of Fay and Feuer (9) to construct interval estimates for ratios based on *F* distributions. Tiwari et al (10) modified this work to yield more efficient interval estimation for rates and ratios of rates from nonnested regions, work that was later extended by Tiwari et al (12) for when one subregion is nested within a larger region (eg, a county nested within a state); Zhu et al (13) extended these approaches to more accurately account for spatial autocorrelation. When the age-standardized rates must be estimated from suppressed data, further modifications must be made or these approaches will fail to adequately account for all sources of uncertainty, yielding interval estimates that may be too narrow (14,15).

Rather than develop the statistical theory to accurately account for substitution-based approaches to overcome CDC WONDER’s privacy restrictions in variance calculations, we consider the use of Bayesian statistical models, which rely on data augmentation to make inference on the suppressed counts. As described by Fridley and Dixon (14), data augmentation approaches estimate the suppressed counts via multiple imputation (16) while simultaneously making inference on the parameters of interest — for example, λ*_ik_* and the effects of potential risk factors. As noted by Zhu et al (13), Bayesian methods for modeling spatial data (17) can yield improved rate estimates when data are limited while simultaneously providing a mechanism for estimating uncertainty in rate estimates — uncertainty that can be seamlessly propagated into estimates such as age-standardized rates and rate ratios. That said, a key drawback of Bayesian methods is their tendency to rely on computationally burdensome Markov chain Monte Carlo (MCMC) methods.

The objective of this analysis was to illustrate 2 Bayesian approaches for estimating county-level mortality rates, by using heart disease mortality data from 1980 obtained from CDC WONDER (18), and to compare these results with those generated by the approach of Tiwari et al (8). In particular, we used a simple, nonspatial Bayesian model, which produces estimates similar to those from Tiwari et al (8), along with a more complex Bayesian model that accounts for spatial and between-age sources of dependence.

## Methods

The study population for this analysis included all residents of the contiguous United States aged 35 or older during 1980. These data have multiple advantages. Because these data were collected before CDC’s suppression guidelines (2) went into effect, the public-use data are complete and free of suppression. Furthermore, because county definitions changed in several ways during the 1980s, the choice of data from 1980 allowed use of readily available shapefiles from the US Census Bureau for the *I* = 3,109 counties (or county equivalents) in the contiguous United States. To replicate the analysis of Tiwari et al (8), the data were separated into *K* = 6 groups: those aged 35 to 44, 45 to 54, 55 to 64, 65 to 74, 75 to 84, and 85 or older. Annual counts of heart disease–related deaths per county per age-group were obtained via CDC WONDER (18) and were defined as those for which the underlying cause of death was “diseases of the heart” according to the *International Classification of Diseases, Ninth Revision* (codes 390–398, 402, 404–429). Of the more than 18,000 counts in this data set, nearly half were fewer than 10.

### Statistical model

Recall that *y_ik_* and *n_ik_* denote the number of deaths and the population size in age group *k* in county *i*. To model these data, we considered 2 approaches: a simple Poisson-gamma model and a multivariate spatial Bayesian model. Although the former illustrates how a Bayesian model with weakly informative priors can produce estimates similar to those obtained directly from the raw data — but with accurate uncertainty measures — the latter illustrates how Bayesian models can incorporate complex dependence structures to produce more reliable estimates. A formal definition of what constitutes a “reliable” rate and the implications of this definition are provided in the Web Appendix (https://sites.google.com/site/harryq/wonder). Because of the complexity of Bayesian models, the Web Appendix also provides technical details on the methods described in this article and includes R (19) and WinBUGS (20) code.

#### Poisson-gamma model

Following the advice of Brillinger (21), we assumed

$y_{ik}|\lambda_{ik}\sim Pois\left(n_{ik}\lambda_{ik}\right)$ (Equation 2)

for *i* = 1, . . ., *I* and *k* = 1, …, *K*. Because we wished to fit Equation 2 using a Bayesian framework, we had to specify a prior distribution for each λ*_ik_*. A convenient choice was to let

$\lambda_{ik}\sim Gam\left(y_{s_{i}0k},n_{s_{i}0k}\right)$ (Equation 3)

As described in the Web Appendix, $y_{s_{i}0k}$ can be interpreted as the prior number of events and $n_{s_{i}0k}$ as the prior population size, thereby providing a mechanism for comparing the informativeness of the prior to the amount of information contained in the data. For example, a prior with $n_{s_{i}0k}=$ 1,000 would contain the same amount of information as the data when *n_ik_* = 1,000, and the posterior mean would be equal to the average of $\lambda_{s_{i}0k}=y_{s_{i}0k}\;/n_{s_{i}0k}$ (the estimate from the prior) and ${\hat{\lambda }}_{ik}=y_{ik}/n_{ik}$ (the estimate from the data). Here, we can take an empirical Bayesian approach by letting $\lambda_{s_{i}0k}={\overset{-}{\lambda }}_{s_{i}0k}$ from Equation 1 and defining the informativeness of the prior to be such that $\sum_{k}y_{s_{i}0k}=$ 6 for all states under the restriction that the $n_{s_{i}0k}=y_{s_{i}0k}/\lambda_{s_{i}0k}$ parameters respect the age distribution in the United States. To better accommodate low rates among the younger age groups, which produce a preponderance of zero counts, we modified the prior in Equation 3 based on the suggestion of Kerman (22) by letting

$\lambda_{ik}\sim Gam\left(y_{s_{i}0k}+1/3,n_{s_{i}0k}\right)$ (Equation 4)

This prior specification can be considered relatively noninformative because 96.4% of US counties had more than $\sum_{k}\left(y_{s_{i}0k}+1/3\right)=$ 8 heart disease–related deaths in 1980. A more complete discussion of this model is provided in the Web Appendix.

#### Multivariate conditional autoregressive model

Although the prior specification in Equation 4 is a convenient choice, it does not take full advantage of the possibilities of Bayesian modeling. In particular, Equation 4 does not account for spatial relationships or the relationships between different age groups. To allow for such structures to be included in the model, we considered Poisson regression models, where

$\log\lambda_{ik}=x_{ik}^{T}\beta_{k}+\theta_{ik}$ (Equation 5)

Here, ***x****_ik_* denotes a vector of county-specific covariates with corresponding age-specific regression coefficients, **β***_k_*; for example, including state-level effects could help account for important health policy differences across state lines. For this analysis, we simply assumed $x_{ik}^{T}\beta_{k}=\;\beta_{0k}$; that is, a model with age-specific intercept parameters. To account for spatial and between-age sources of dependence, we first followed the approach of Besag et al (17) and defined $\theta_{ik}=z_{ik}+\varphi_{ik}$, where *z_ik_* accounts for spatial structure within each age-group and $\varphi_{ik}$ denotes an exchangeable (ie, nonspatial) random effect. More specifically, the conditional autoregressive (CAR) model of Besag et al (17) imposes spatial structure by shrinking each *z_ik_* toward the values in neighboring counties (ie, counties that share a border), where the strength of this shrinkage is controlled by the number of neighboring counties.

Although the CAR model is a powerful tool for analyzing spatial data, it does not account for possible correlation between the multiple age groups. To account for this, we instead considered a multivariate extension of the CAR model: the multivariate CAR (MCAR) model of Gelfand and Vounatsou (23). As with the CAR model, the MCAR shrinks estimates toward their neighboring values; unlike the CAR model, however, the MCAR explicitly models the between-group correlation in the data and leverages these correlations to produce more precise age-specific rate estimates. MCAR models were used recently to model spatially referenced survival times in cancer data (24), temporal trends in county-level asthma hospitalization rates (25), temporal trends in heart disease mortality by race and sex (26), and temporal trends in age-specific stroke mortality (27), among many other applications. Full details, including a discussion of the prior distributions used, are provided in the Web Appendix.

#### Bayesian inference

Fitting the models in Equation 4 or Equation 5 while accounting for the suppression of counts fewer than 10 requires the use of MCMC algorithms. Because of the reliance on MCMC, inference from these Bayesian models is based on samples generated from the posterior distribution — for example, $\lambda_{ik}^{\left(l\right)}$ for *l* = 1, …, *L*, where *L* denotes the number of samples. These samples can then be used to compute quantities such as the age-standardized mortality rate:

$\lambda_{i∙}^{\left(l\right)}=\sum_{k}\pi_{k}\lambda_{ik}^{\left(l\right)}$

where π*_k_* denotes a prespecified standard age distribution (eg, based on the 2010 US standard population). To summarize the posterior distribution, it is common to use the posterior median and the 95% credible interval (constructed from the 2.5 and 97.5 percentiles of the posterior samples and analogous to classical 95% confidence intervals).

### Comparison of approaches

To compare the various estimation approaches, we first considered simple correlations between the estimates and the rates obtained from the complete data (as considered by Tiwari et al [8]) and correlations between the age-standardized rates and the age-specific rates. The goal of these comparisons was not to demonstrate whether one approach is superior to another but rather to demonstrate the degree to which the approaches are similar to one another. In addition, we also compared the 2 Bayesian approaches by using the deviance information criterion (DIC) (28), which uses the posterior samples to produce a measure that is a compromise between model fit (denoted by $\overset{-}{D}$) and model complexity, *p_D_*. In particular, *p_D_* is often interpreted as the effective number of parameters in the model. Additional details on DIC, including a discussion of its use with censored data, are provided in the Web Appendix.

### Creation of maps

Maps were created by using the R statistical software (The R Foundation). Code is available in step 6 of the walkthrough in the Web Appendix (https://sites.google.com/site/harryq/wonder).

## Results

**Figure 1:**
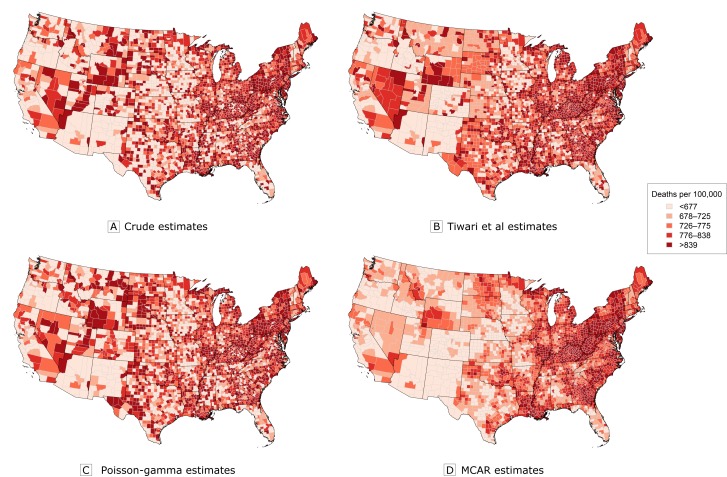
Estimates of age-standardized heart disease mortality rates from 1980. A, Crude age-standardized rates based solely on the data. B, Estimates obtained by using the approach of Tiwari et al (8). C, Estimated posterior medians from the Poisson-gamma model. D, Estimated posterior medians from the multivariate conditional autoregressive model (MCAR). Data source: Centers for Disease Control and Prevention (18).

The maps of the age-standardized rates generated from the raw data (Figure 1A) and the maps generated by the Poisson-gamma model (Figure 1C) have strong similarities, while artifacts of substituting state-wide averages for suppressed counts based on the approach of Tiwari et al (8) lead to elevated estimates in many rural counties in the upper Midwest (Figure 1B). In contrast, the map of the estimates from the MCAR model (Figure 1D) preserves the overall trends in the data while producing significantly smoother rate estimates.

**Figure 2:**
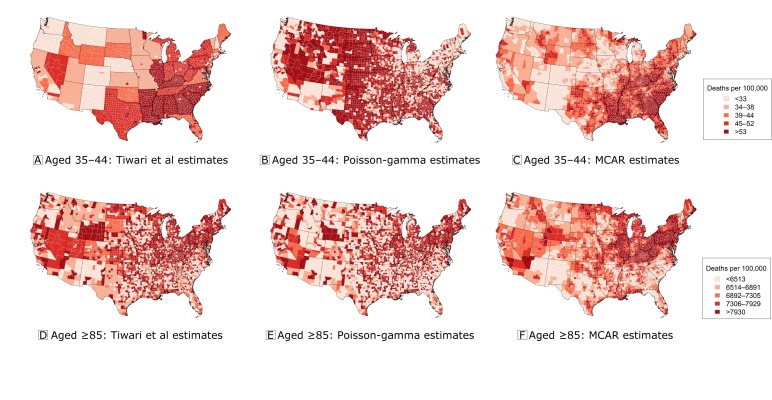
Comparison of 3 approaches for estimating age-standardized heart disease mortality rates for 2 age groups (adults aged 35 to 44 and adults aged ≥85) from 1980. A, Estimates for adults aged 35 to 44 obtained by using the approach of Tiwari et al (8). B, Estimated posterior medians for adults aged 35 to 44 from the Poisson-gamma model. C, Estimated posterior medians for adults aged 35 to 44 from the multivariate conditional autoregressive model (MCAR). D, Estimates for adults aged ≥85 obtained by using the approach of Tiwari et al (8). E, Estimated posterior medians for adults aged ≥85 from the Poisson-gamma model. F, Estimated posterior medians for adults aged ≥85 from the multivariate conditional autoregressive model (MCAR). Data source: Centers for Disease Control and Prevention (18).

The correlation results (Table 1) largely support this assessment. The Poisson-gamma approach produced age-standardized rate estimates that were the most highly correlated with the true rates, although the estimates obtained by using the substitution approach of Tiwari et al (8) had nearly an identical correlation. These 2 approaches differed in age-specific rate estimates. In particular, although the Poisson-gamma approach appeared to struggle for adults aged 35 to 44 — producing estimates that were less correlated with the truth — it outperformed the substitution approach for all groups aged 55 or older. Figure 2, which displays the age-specific rate estimates for adults aged 35 to 44 and adults 85 or older, explains how this occurred. Here, although the approach of Tiwari et al (8) gave every suppressed county in each state the same rate (by design), the Poisson-gamma model tended to overestimate rate estimates for those aged 35 to 44. According to Kerman (22), this overestimation of rates when counts are very small was to be expected. Furthermore, unlike the approach of Tiwari et al (8), the Poisson-gamma model produced full posterior distributions for each age-specific rate estimate, thereby allowing quantification of the uncertainty in these estimates. (Figure B.3 in the Web Appendix illustrates how only 4.5% of estimates for those aged 35 to 44 and 42.8% of all age-specific rate estimates from the Poisson-gamma model were deemed reliable.) When estimating rates for those 85 or older, the Poisson-gamma model permitted heterogeneity within states (Figure 2E); the inability to permit such heterogeneity is a key weakness of the approach of Tiwari et al (8). Further evaluation of the low age-specific correlations is provided in the Web Appendix (Figures B.1 and B.2).

**Table 1:** Comparison of the Correlation Results of 3 Estimation Approaches, Analysis of County-Level Mortality Rates Using Highly Censored Data From CDC WONDER^a^

Approach	Age Group						Age-Standardized
	35–44	45–54	55–64	65–74	75–84	≥85	
Tiwari et al (8)	0.15	0.73	0.16	0.07	−0.01	0.08	0.73
Poisson-gamma	0.09	0.74	0.23	0.25	0.24	0.27	0.74
Multivariate conditional autoregressive model	0.15	0.65	0.18	0.15	0.05	0.14	0.65

a Age-standardized correlation results were based on all 3,109 US counties, whereas age-specific correlation results were based only on the suppressed counties (counties with counts <10). Data source: Centers for Disease Control and Prevention (18).

Looking at the correlation results (Table 1) and the maps in Figure 1, one may wonder why we bother fitting the complex MCAR model. The DIC results (Table 2) explain why. Here, the MCAR model offered a model fit that is similar to the fit of the Poisson-gamma model (as measured by $\overset{-}{D}$) while doing so with far fewer “effective model parameters” (*p_D_*). To understand how this can be, recall that each λ*_ik_* in Equation 4 had its own independent prior distribution; that is, the Poisson-gamma model did not shrink the λ*_ik_* toward each other, producing estimates of the (*p_D_*) for older age groups that approach the full *I* = 3,109 number of parameters. In contrast, the MCAR model explicitly imposed dependence between its model parameters, resulting in estimates of the (*p_D_*) that were nearly 80% less than those from the Poisson-gamma model (eg, 10,785 vs 2,307). In addition, the estimates produced by the MCAR model were more precise (Web Appendix), and the smooth geographic patterns in Figure 1D, Figure 2C, and Figure 2F may provide clearer insight into the underlying trends in heart disease mortality.

**Table 2:** Comparison of the Deviance Information Criterion^a^ Results of 3 Estimation Approaches, Analysis of County-Level Mortality Rates Using Highly Censored Data From CDC WONDER^b^

Approach	Age Group						Overall
	35–44	45–54	55–64	65–74	75–84	≥85	
**Poisson-gamma**							
DIC	2,204	6,108	12,393	17,866	19,005	16,956	74,533
$\overset{-}{D}$	1,663	5,006	10,509	15,447	16,506	14,616	63,748
*p_D_*	542	1,102	1,884	2,419	2,499	2,339	10,785
**Multivariate conditional autoregressive model**							
DIC	1,558	5,242	11,245	16,201	17,417	15,904	67,568
$\overset{-}{D}$	1,478	5,030	10,842	15,743	16,887	15,281	65,260
*p_D_*	80	213	403	458	530	624	2,307

a Spiegelhalter et al (28).

b Where $\overset{-}{D}$ is a measure of model fit (lower is better), *p_D_* is a measure of model complexity (lower indicating fewer effective model parameters), and $DIC=\overset{-}{D}\;+p_{D}.$. Data source: Centers for Disease Control and Prevention (18).

## Discussion

This analysis highlighted some of the benefits of using Bayesian methods to account for left-censored data like those encountered in CDC WONDER. Although the Poisson-gamma model is a relatively simple approach, models (such as the MCAR model) that explicitly account for multivariate spatial dependence structures can lead to better inference by leveraging other sources of information to produce more reliable estimates.

The strengths of the MCAR model described in this analysis extend beyond modeling censored data to the broader field of small area estimation. As alluded to in the discussion of Equation 5, many benefits are associated with using the MCAR model in conjunction with covariate information when modeling chronic disease outcomes. Combining covariate information with spatial structure can produce more reliable estimates of the rates themselves, which is beneficial for disease surveillance, while simultaneously conducting inference on the potential risk factors that are included as covariates. When the covariates in the analysis are themselves spatially structured, it can be unclear if the covariate is effecting change in the outcome or vice versa, or if an unmeasured spatial confounder is influencing both the covariate *and* the outcome. In these settings, including a spatial random effect can lead to a phenomenon referred to as “spatial confounding” (29) and increase the standard errors associated with these covariates. Although the notion of spatial confounding has historically been considered a drawback of spatial models (29), others have argued (30) that inference from such models can help protect against type 1 error (ie, incorrectly rejecting the null hypothesis).

Finally, although we analyzed age-specific heart disease mortality as an illustration, the MCAR model is also well suited for analyzing rarer event data via its ability to jointly model multiple outcomes. This analysis leveraged information from older age groups with higher death counts to produce more reliable estimates for those aged 35 to 44. Similarly, one could jointly model a chronic disease outcome for multiple race/ethnicities, exploiting the shared factors that may lead to increased rates for non-Hispanic white persons and racial/ethnic minorities alike. Alternatively, one could use MCAR models to simultaneously analyze multiple chronic disease outcomes with similar etiologies to improve the reliability of all estimates.

Although the suppression of data creates an obstacle to conducting chronic disease surveillance, Bayesian statistical methods such as those described in this analysis can overcome these challenges while also producing more reliable estimates with valid uncertainty measures. By illustrating the benefits of and providing code for their implementation, we hope to ease the burden of using Bayesian models and broaden their application to censored data sets available from sources like CDC WONDER, thereby improving the inference made from public-use data.
